# Associations between School-Level Disadvantage, Bullying Involvement and Children’s Mental Health

**DOI:** 10.3390/children10121852

**Published:** 2023-11-25

**Authors:** Julia R. Badger, Mirela Zaneva, Richard P. Hastings, Matthew R. Broome, Rachel Hayes, Paul Patterson, Naomi Rose, Suzy Clarkson, Judy Hutchings, Lucy Bowes

**Affiliations:** 1 Department of Experimental Psychology, University of Oxford, Oxford OX2 6GG, UK; julia.badger@education.ox.ac.uk (J.R.B.);; 2 Department of Education, University of Oxford, Oxford OX2 6PY, UK; 3 Christ Church College, University of Oxford, Oxford OX1 1DP, UK; mirela.zaneva@chch.ox.ac.uk; 4 School of Education Learning and Communications Sciences, University of Warwick, Coventry CV4 7AL, UK; r.hastings@warwick.ac.uk; 5 Institute for Mental Health, University of Birmingham, Birmingham B15 2TT, UK; m.r.broome@bham.ac.uk; 6 Birmingham Women’s and Children’s NHS Foundation Trust, Birmingham B4 6NH, UK; paul.patterson2@nhs.net; 7 Department of Public Health and Sports Sciences, Faculty of Health and Life Sciences, University of Exeter, Exeter EX1 2LU, UK; r.a.hayes@exeter.ac.uk; 8 School of Human and Behavioral Sciences, Bangor University, Bangor LL57 2AS, UK; s.clarkson@bangor.ac.uk (S.C.); j.hutchings@bangor.ac.uk (J.H.)

**Keywords:** bullying perpetration, victimization, disadvantage, mental health, emotional symptoms, externalizing problems

## Abstract

Bullying is a modifiable risk factor for poor mental health across childhood and adolescence. It is also socially patterned, with increased prevalence rates in more disadvantaged settings. The current study aimed to better understand whether school-level disadvantage is associated with different types of bullying roles, and whether it is a moderator in the association between bullying and children’s mental health. Cross-sectional data were used from 4727 children aged 6–11 years, from 57 primary schools across England and Wales. The child data included previous bullying involvement and bullying role characteristics (bully, victim, bully–victim, reinforcer, defender, outsider), and the teacher-reported data included each child’s mental health (emotional symptoms and externalizing) problems. School-level disadvantage was calculated from the proportion of children in the school eligible to receive free school meals (an indicator of disadvantage). Children in more disadvantaged schools were more likely to report being bully perpetrators, bully–victims, and engage less in defending behaviors during a bullying incident. Children from more disadvantaged schools who reported bullying others showed fewer emotional symptoms than those from less disadvantaged schools. There was no other evidence of moderation by school-level disadvantage between bullying roles and emotional and externalizing problems. The findings highlight the potential for school-based interventions targeting children’s emotional and social development, targeting bullying, and promoting defending behaviors, particularly in more disadvantaged settings.

## 1. Introduction

Mental health in children and adolescents is a public health priority. A recent UK longitudinal study showed that the probable rates of mental disorders for children aged 6–16 years had risen from 11% in 2017 to 17% in 2021 and 18% in 2022 [1]. The findings are mirrored by data from the American Centre for Disease Control and Prevention (CDC) report that in 2022, 20% of children and young people in America had an identified mental health disorder [2].

### 1.1. Bullying and Mental Health

Bullying is a damaging and aggressive repeated behavior characterized by an imbalance of power between the perpetrator(s) and the targeted victim. The intention of the interaction is to cause the victim harm. School bullying is a known modifiable risk factor associated with children’s worsened mental health and negative consequences that can remain into adulthood [3,4,5].

For many years, the research focused on the involvement of the bully and the victim in a bullying situation and on the associations with mental health [3]. Other roles that children take on during a bullying incident have since been identified, including ‘assistants’, ‘reinforcers’, ‘defenders’, and ‘outsiders’ [6,7]. Those children surrounding the bullying incident are now recognized as playing a crucial role in the initiation and maintenance or reduction in bullying behaviors; therefore, all roles will be included in this paper.

Bullying victimization and perpetration have both been described as negative environmental stressors that might contribute to poor mental health, particularly among those with greater vulnerability [8]. The complex group of individuals known as ‘bully–victims’ who both bully others and are victimized themselves are particularly vulnerable due to their double-role and are at risk of psychological problems [9,10]. The evidence also suggests that children involved in the wider participant roles in bullying may, as a result, be at increased risk of developing mental health problems. For example, children who witness bullying but do not (or perhaps cannot) intervene—known as ‘outsiders’—may also experience heightened emotional and behavioral problems [11]. ‘Reinforcers’ of bullying, on the other hand, may experience greater levels of cognitive dissonance as they struggle to balance the need to protect themselves with the knowledge that they are helping those bullying others [12]. Such cognitive dissonance can lead to feelings of self-blame and anger, increasing the risk of both internalizing (emotional difficulties including symptoms of anxiety and depression) and externalizing (behavioral difficulties including conduct problems, hyperactivity, and aggressive behaviors) problems. It is important, therefore, to consider the involvement and outcomes for all roles within a bullying situation.

### 1.2. Bullying and Disadvantage

Understanding contexts that exacerbate or attenuate the link between bullying and children’s mental health is crucial. One such context may be school-level disadvantage—the concentration of child-level disadvantage within a school. With children spending so many hours each week in school, schools can be seen as a very influential (either positive or negative) environment on a child’s development [13,14]. They are being recognized as microcosms that can either foster positive development or can host adversity, places designed to support learning and knowledge acquisition, and also places that nurture children’s social development and health. School bullying involvement can, therefore, create a negative experience within a child’s microsystem.

Bullying is more frequently experienced by children growing up in more disadvantaged homes and communities (see [15] for a review); for example, a meta-analysis [16] found that children from the lowest income households had 40% higher odds of experiencing bullying compared to children from the highest income households. There are also socio-economic patterns that account for some of the extensive variation in prevalence rates of bullying between schools [17]. Currently, it remains unclear as to whether school-level disadvantage moderates the association between bullying involvement and mental health problems in children.

Schools with a higher concentration of disadvantage are known to be associated with increases in school-based violence and disorder, poorer academic outcomes, and an overall worsened school climate and environment [18,19]. They may have fewer resources to implement anti-bullying programs or provide adequate supervision, potentially fostering a climate where bullying can thrive. Furthermore, pupils in such schools may be more likely to engage in bullying behavior as a means of coping with their own stressors. Therefore, although limited research has considered whether the presence of disadvantage within a school can exacerbate the prevalence of bullying, regardless of individual home and community levels of disadvantage, it seems possible. These problems can lead to disruptions in the learning environment and negative long-term consequences for children’s social and academic development. Therefore, understanding the association between school-level factors, including the concentration of disadvantage, and mental health outcomes can provide valuable insight into how schools can begin to support and improve children’s mental health.

### 1.3. Current Study

We used data from over 4000 UK primary school children aged 6–11 years from 57 schools to investigate whether school-level disadvantage (the concentration of disadvantaged pupils in a school) moderated the association between bullying involvement and mental health (externalizing and emotional) problems in children. We administered the Olweus Bullying and Victimization Questionnaire (OBVQ) and the Participant Role Questionnaire (PRQ) to children to identify their involvement in bullying and bullying roles (bully, victim, bully–victim, reinforcer, defender, outsider) and the Strengths and Difficulties Questionnaire (SDQ) to identify the teacher-rated levels of externalizing and emotional problems of each child. Understanding contexts that exacerbate or attenuate the link between bullying and children’s mental health is an important goal for the development and targeting of anti-bullying interventions.

We focused on addressing the following research questions:

1. Was the level of bullying involvement higher when the level of school-level disadvantage was higher as determined by the proportion of children eligible for ‘free school meals’?
2. Was bullying involvement (including reinforcing, defending, and outsider roles) associated with increased levels of mental health (emotional and externalizing) problems?
3. Does school-level disadvantage moderate any associations between bullying involvement and children’s mental health (emotional and externalizing) problems?

## 2. Materials and Methods

### 2.1. Design

A cross-sectional design was used. Data were collected between February and March 2020 as part of the Stand Together Trial, a randomized-controlled trial examining the effectiveness of the KiVa anti-bullying intervention [20]. Schools had not been assigned a trial condition at the time of baseline data collection and all data were collected pre-COVID-19 pandemic. Ethical approval was granted by the University of Bangor Ethics and Research Committee.

### 2.2. Participants

A total of 4724 children from 57 primary schools in England (*n* = 36) and Wales (*n* = 21) participated. Schools were sampled from four geographic regions (Devon, Oxfordshire, West Midlands, North Wales). Strategic postcode sampling was applied in an attempt to include a representative range of schools within each region.

UK primary schools are for children of approximately 4–11 years of age. All children in school years 3 (age 7–8), 4 (age 8–9), and 5 (age 9–10) from these schools were invited to participate; parents could opt their children out of the study and the remaining children assented on the day of data collection (sex: female = 48.5%; mean age = 8 years 6 months (SD = 0.97 years): age 6 = 0.1%; age 7 = 15.6%; age 8 = 33.1%; age 9 = 32.2%; age 10 = 18%; age 11 = 0.1%; age data missing from 0.8%). The schools’ year 3–year 5 cohorts ranged in size from 28 to 277 children (*M* = 100.7; SD = 61.63). School size was based on the number of children registered in those years, regardless of whether they decided to take part in the study. Eight children did assent to take part initially but did not complete any questionnaire data and were removed from all analyses.

### 2.3. Measures

#### 2.3.1. School-Level Disadvantage

This study focused on school-level disadvantage defined as the percentage of children in a school eligible to receive free school meals (eFSM). In the early 1990s, the UK Government recognized the benefit of providing a daily free school meal to the most disadvantaged pupils in the country aged 5 to 16 years. They introduced the Education Act 1996, which required schools to provide daily free school meals to any child whose parents were in receipt of certain income-determined government benefits, an indicator of socio-economic deprivation. The national average of children eFSM at the time of the research was 17.3% in English primary schools [21] and 18.8% in Welsh primary schools [22], and was 14.85% (SD = 13.56) across the schools in the current study. School-level disadvantage (the percentage of children eFSM in each school) was used as a continuous variable for the statistical analysis (with the exception of the analyses for our first question, where we preregistered models with both the continuous and categorical variable and the categorical variable was created based on a median split of our school-level eFSM data).

#### 2.3.2. Olweus Bullying and Victimization Questionnaire (Bullying Involvement)

We administered the Olweus Bullying and Victimization Questionnaire (OBVQ) [23] to categorize each child’s bullying involvement roles into one of the four possible categories: bully, victim, bully–victim, and not involved. Questions were scored 1–5, where 1 indicated it had not happened, 3 indicated 2 or 3 times a month, and 5 indicated several times a week. Twenty-two questions were asked, of which 20 were used to create the dichotomous variables used in our analyses. Ten questions indicated having bullied someone (e.g., “How often have you taken part in bullying another child at school in the past couple of months?” and “I called another child mean names, made fun of, or teased them in a hurtful way”) and 10 indicated having been bullied (e.g., “How often have you been bullied at school in the past couple of months?” and “I was called mean names, made fun of, or teased in a hurtful way”). For categorization purposes, we followed the literature [24], whereby a child had to answer ‘2 or 3 times a month’ or more often to at least one of the 10 bully perpetration questions to be classified as a perpetrator of bullying. The same rule was applied when categorizing a victim from the 10 victimization questions. To be classified as a bully–victim (an individual who both perpetrates bullying and is bullied themselves), a child had to answer ‘2 or 3 times a month’ or more often on at least one of the 10 bully perpetration questions and one of the 10 victimization questions. Children that never answered ‘2 or 3 times a month’ or more often on any of the 20 questions were classified as ‘not involved’. For our analyses with the OBVQ classification, we excluded individual participants who had missing data or who had responded with the ‘prefer not to say’ option on 50% or more of the OBVQ questions about being bullied and 50% or more of the OBVQ questions about bullying others. After considering practical guidelines regarding missing data [25], we applied the 50% criterion to be able to make rigorous classifications whilst still maximizing the number of participants we could include. Following this procedure, we created dichotomous variables for each category (bully, victim, bully–victim, not involved), whereby we recorded whether a child was a member of this category. Given the classification method, these categories were exclusive (children could not be in more than one group) (internal consistency: α = 0.91 (victimization) and α = 0.87 (perpetration)).

#### 2.3.3. Participant Role Questionnaire (Bullying Roles)

The Participant Role Questionnaire (PRQ) [26] was administered as an extended measure of bullying role classification, including roles beyond the bully and victim (bully perpetrator, assistant, reinforcer, defender, and outsider). Due to a survey setup error, we did not collect data on the assistant role scale. Thus, the PRQ that was collected comprised 12 questions, with three questions corresponding to each of four role classifications. The PRQ questions were adapted from the original peer reporting method to self-reporting. The questions were scored as follows: 0—never; 1—sometimes; 2—often. The scores were summed for each scale, resulting in a total score for each role (e.g., for defenders), with a possible range of 0–6, where higher scores indicate more frequent involvement in the behaviors corresponding to that role. For the PRQ analyses, if a participant had not responded to all three questions within each scale, we were not able to compute their score for that corresponding scale [26]. As long as a participant replied to at least one scale in full, we included them in the corresponding analyses (internal consistency: α = 0.64 (bully), α = 0.72 (defender), α = 0.13 (reinforcer), and α = 0.03 (outsider)).

#### 2.3.4. Strengths and Difficulties Questionnaire

The Teacher Strengths and Difficulties Questionnaire (TSDQ) [27] was completed by class teachers as a measure of each child’s emotional and behavioral problems. The TSDQ has twenty-five questions, of which 15 were used in our analyses; five comprised the emotional symptoms score, and the 5 questions measuring conduct problems and hyperactivity and inattention were combined to make an externalizing problem score. The peer relationship subscale was not included (which would typically have been combined with the emotional symptoms score to make an internalizing score) due to potentially confounding with our bullying measures. Each question was scored from 0 (not true) to 2 (certainly true) and the question scores were summed for each scale, with higher scores indicating greater levels of problems. Here, data were missing from seven schools; therefore, these schools were not be included in any TSDQ analyses (internal consistency: α = 0.85 (emotional) and α = 0.88 (externalizing)).

### 2.4. Procedure

The researchers attended each class for one hour and read through and explained every question to the children. The children completed the OBVQ and PRQ in one session on electronic tablets. The class teachers completed the TSDQ for each of their students on paper questionnaires.

### 2.5. Analysis Plan

Our analyses were preregistered at https://osf.io/xk2vm (19 August 2021). We first examined in our sample the numbers of victimized, bully perpetrators, and bully–victims in more disadvantaged versus less disadvantaged schools. Next, we examined whether bullying involvement was associated with increased teacher-reported levels of emotional and externalizing problems. We used multilevel regression and clustering by school, whereby our outcome variables, emotional and externalizing problems, were computed based on the TSDQ scores. Finally, we investigated whether school-level disadvantage moderated the association between bullying involvement and children’s emotional and externalizing problems. We used multilevel regression accounting for clustering by school, with emotional and externalizing problems as the outcome variables. For predictors, we examined bullying involvement, school-level eFSM proportion, and their interaction. Additionally, we examined the same research questions using data on bullying roles from the PRQ in place of bullying involvement variables. Our analyses with the PRQ were exploratory. All analyses were carried out in R version 4.0.5. We relied on the package nlme to run our regressions with the REML estimator.

## 3. Results

We note that although our full sample contained data from 4724 students, our analyses were based on fewer observation (max: 4258; min: 2640). This was due to missing data and the use of pairwise deletion, with the numbers of missing responses differing depending on the specific sub-scale or measures. We ran Little’s test to examine whether our OBVQ, PRQ, and TSDQ data were missing completely at random. All three tests were significant at *p* < 0.0001, suggesting that data were not missing completely at random. Thus, it remains possible that the data were missing at random (MAR) or missing not at random (MNAR).

### 3.1. Bullying Roles and School-Level Disadvantage

The average proportion of children across all schools reporting victimization was 36.22 ± 8.87%, reporting bully perpetration was 2.01 ± 1.61%, reporting being bully–victims was 11.01 ± 6.81%, and reporting being not involved in bullying was 51.16 ± 10.35%. The mean PRQ bully perpetration across all children score was 0.32 ± 0.17, the mean reinforcer score was 1.34 ± 0.17, the mean defender score was 4.58 ± 0.37, and the mean outsider score was 2.66 ± 0.23.

The association between bullying involvement and the degree of school-level disadvantage was explored in a multilevel model, where individual-level OBVQ bullying involvement rates were the predictor variable (separate models for victimized, bully perpetrator, bully–victim, and not involved) and school-level disadvantage was the outcome variable. A random intercept at the school level in each model was included to account for the clustering of individuals within schools (see Table 1). The proportion of bully–victims was higher in schools with a greater level of disadvantage (% school-level eFSM) when this was assessed as a continuous measure (B = 0.002, *p* < 0.001) and as a dichotomized (median split of our school-level) measure (B = 0.03, *p* < 0.05). These analyses did not indicate significant associations between school-level disadvantage (continuous or dichotomized) with bullying perpetration and indicated only a significant association between victimization and school-level disadvantage as a dichotomized measure (B = −0.04, *p* < 0.05; see Table 1).

We investigated the association between the PRQ scores and school-level deprivation via unadjusted multilevel regression analyses. The PRQ mean scores for different bullying roles (perpetrator, reinforcer, defender, outsider) were the outcome variables in each model and school-level disadvantage was the predictor measure. Again, in each model, we included a random intercept at the school level to account for the clustering of individuals within schools (see Table 2). We found that in schools with more disadvantage, children were less likely to engage in defending behaviors (B = −0.01, *p* < 0.05). This was also true when using a binary predictor for school-level disadvantage (B = −0.25, *p* < 0.01). We also found that children were more likely to engage in bullying perpetration when from a school with more disadvantage (B = 0.003, *p* < 0.05).

### 3.2. Bullying Involvement, Mental Health, and Moderation by School-Level Disadvantage

Through a series of multilevel regressions with clustering accounted for at the school level, we examined the associations between bullying involvement, teacher-rated emotional symptoms (Table 3), and externalizing problems (Table 4). We also tested whether the associations were moderated by disadvantage at the school level. The tables report on the unadjusted and adjusted (for age, sex, school-level eFSM, and interactions) models.

#### 3.2.1. Emotional Symptoms and the OBVQ

As expected, we found that children reporting their involvement as victims or bully–victims had increased levels of emotional symptoms compared to children who reported no bullying involvement (B = 0.30, *p* < 0.001 and B = 0.46, *p* < 0.001, respectively; see Table 3).

Our data also suggest that children in schools with a greater level of disadvantage are generally more likely to display emotional symptoms (B = 0.02, *p* = 0.016) independent of their involvement—if any—in bullying. This was still true in our adjusted model (B = 0.02, *p* = 0.002). Children self-reporting bullying perpetration behaviors from more disadvantaged schools were reported to have significantly fewer emotional symptoms (B = −0.05, *p* = 0.017); this was still true in our adjusted model (B = −0.06, *p* = 0.01).

#### 3.2.2. Externalizing Problems and the OBVQ

As expected, children reporting their involvement as bullies, victims, and bully–victims had increased levels of externalizing problems compared to children who reported no bullying involvement (B = 1.81, *p* < 0.001; B = 0.42 *p* < 0.001; B = 1.61, *p* < 0.001, respectively, see Table 4) in both unadjusted and adjusted models. Our data also show that children in schools with a greater level of disadvantage were not more likely to display externalizing symptoms in our unadjusted model (B = 0.01, *p* = 0.072), although this became significant in our adjusted model (B = 0.01, *p* = 0.02). School-level disadvantage did not moderate the association between bullying involvement (as a bully, victim, or bully–victim) and externalizing problems in either our unadjusted or adjusted models.

Through a further series of multilevel regressions clustered at the school level, we examined the association at the individual level between bullying role behaviors (perpetrator, reinforcer, defender, and outsider behavior scores) and children’s emotional problems (Table 5) and externalizing problems (Table 6).

#### 3.2.3. Emotional Symptoms and the PRQ

Those children reporting more defending behaviors were overall reported by their teachers to have lower levels of emotional symptoms (B = −0.08, *p* = 0.007; see Table 5). Children reporting reinforcing behaviors were found to have higher levels of teacher-reported emotional symptoms in the adjusted model (B = 0.17, *p* < 0.05), although no interaction was found. Our adjusted models suggest that older children and girls reported higher levels of emotional symptoms (B = 0.11, *p* = 0.023; B = 0.32, *p* < 0.001, respectively). We did not find support for a direct or moderating effect of school-level deprivation on the association between PRQ scores and emotional symptoms.

#### 3.2.4. Externalizing Problems and the PRQ

Those children reporting more bullying behaviors were overall reported by their teachers to have higher levels of externalizing problems (B = 0.60, *p* < 0.001; see Table 6), both in our unadjusted and adjusted models, whereas those children reporting more defending or outsider behaviors were overall reported by their teachers to have lower levels of externalizing problems (B = −0.10, *p* < 0.001; B = −0.11, *p* < 0.001, respectively), both in our unadjusted and adjusted models. We found that girls reported significantly fewer externalizing problems than boys (B = −0.93, *p* < 0.001). We did not find support for a direct or moderating effect of school-level deprivation on the association between PRQ scores and externalizing problems.

## 4. Discussion

This paper aimed to explore the interplay between school-level disadvantage, bullying involvement, and mental health in children from 57 primary schools in the UK. It is well known that children growing up in disadvantaged home life circumstances are more likely to experience bullying [15,16] and that concentrations of school-level disadvantage and community disadvantage are known to be risk factors for school-based violence [18,19,28]. This paper suggests that attending a school with a higher concentration of disadvantage has an association with the amount and type of bullying involvement identified; more disadvantaged schools had children self-reporting higher levels of bully perpetration and bully–victim behaviors. Our results provide further evidence of the negative impact an environment with a high concentration of disadvantage can have on children; the children in our sample from more disadvantaged schools were significantly more likely to have poorer mental health compared to those from less disadvantaged schools, regardless of their involvement, if any, in bullying. Children from more disadvantaged schools were more emotionally insecure, which is perhaps unsurprising considering the schools with a higher concentration of disadvantage included significantly more children who self-reported bullying behaviors and significantly fewer children in those schools reported defending behaviors during a bullying incident. This suggests a negative school environment. Surprisingly, children from more disadvantaged schools who reported bullying others were teacher-reported to also show fewer emotional symptoms than those from less disadvantaged schools. Children reporting more defending or outsider behaviors were reported to have fewer mental health problems.

Knowing that social disadvantage and emotional insecurity are forms of vulnerability and that vulnerable children are more at risk of negative outcomes such as bullying involvement [29,30], it is not surprising that the children in our more disadvantaged schools showed higher rates of bullying involvement. This intensified vulnerability may also go some way to explaining why we found fewer defending behaviors in children from more disadvantaged schools. Defenders in our study and in the wider literature have been shown to have higher levels of emotional stability and prosocial behaviors [31,32]; we propose two possible reasons why fewer children reported defending behaviors in schools with a higher concentration of disadvantage. Firstly, in our study, children in more disadvantaged schools showed higher levels of emotional instability, regardless of their bullying involvement. Children with increased emotional instability and the potential cognitive dissonance of either being or being associated with bullies or bully–victims may experience a suppression of acting upon their prosocial intentions [33]. Secondly, we found that schools with more disadvantage had a significantly higher number of self-reported bullies. Therefore, it is possible that children whose natural tendency would be to defend in a bullying situation find themselves in an environment where bullying is normalized, and those involved are awarded popularity and social status [34]. In those situations, perhaps defending behaviors carry high-risks.

In general, bully perpetrators are more likely to show higher levels of emotional difficulty compared to those not involved in bullying situations [4]. However, we found a moderation of school-level disadvantage, whereby children who self-reported bully perpetration behaviors attending more disadvantaged schools were reported to have fewer emotional instability symptoms compared to self-reported bullies attending less disadvantaged schools. This suggests that social inequality may be influencing the association between bullying and emotional outcomes. Attending a school with higher levels of disadvantage may decrease the association between bullying perpetration and emotional instability. If a school has higher levels of bullying (and higher numbers of bully–victims), then bullying involvement may become trivialized and normalized [34]. Alongside the combined observation of reduced defending behaviors in disadvantaged schools, this suggests that the social positioning and status of bullies may be further strengthened [34]. In turn, the act of being a bully may have less of a negative psychological impact; the disadvantaged school climate may have protected the bullies from increased feelings of guilt, anger, and isolation that can result in emotional symptoms. Children from more disadvantaged homes and communities are also more likely to have experienced bullying behaviors outside the school environment, which might also normalize their bullying involvement within school [16].

### 4.1. Limitations and Future Work

This study included a large sample of primary school children from a range of schools across England and Wales. Although these schools also varied in their level of disadvantage, the study’s percentage of eFSM was a little lower than the national averages in England and Wales, thereby limiting the generalization of the data. We collected data from children and teachers to provide a wider perspective on the situation of, and association between, bullying and mental health. The teachers’ data were concurrent but the children’s data were collected retrospectively via self-report questionnaires. Although the OBVQ timeframe was short (asking for reported bullying experience in school over the past 3 months), this does open up the possibility of recall error by either over- or under-reporting. In addition, it is likely that this study underestimated the prevalence of bullying perpetration due to the self-report measure and the respondents’ fear of being negatively perceived. Due to time restrictions and ethical consideration, we used the PRQ in a self-report format rather than its original peer-reporting format. Future work should consider whether the PRQ as a self-report tool provides the same level of accuracy compared to when it is used as a peer-report tool. We acknowledge that we found poor reliability for the PRQ reinforcer and outsider sub-scales as captured through low Cronbach alpha values. Our analyses with the PRQ were exploratory and further work is needed to clarify or substantiate the effects assessed with the PRQ questionnaire. It is important to point out that the study is unable to identify a causal direction due to the cross-sectional methodology and the inability to include other potentially important variables, including ethnicity and family income, which should be included in the future to explore alternative explanations for the outcomes found.

### 4.2. Conclusions

Our data present the interplay between social inequality, bullying involvement, and mental health. The levels of disadvantage vary across schools in the UK and other countries, which means that we need to make sure that appropriate interventions and support strategies are in place and that they are effective across the social disadvantage spectrum. Gaining a greater understanding of the association between school-level disadvantage and children’s outcomes can help to guide the development of effective and sustainable anti-bullying interventions. Our results suggest the need to focus on encouraging defending behaviors within the most disadvantaged schools and reducing the social positioning and status of bullies. With these changes, it is possible that a more positive school climate would begin to become established. With more research, schools may want to move from individual-child-based interventions and support to fostering a whole-school approach to raise children’s social and emotional wellbeing [7,35].

## Figures and Tables

**Table 1:** Associations between prevalence rates of victimization, perpetration, bully–victims, and not involved (OBVQ) at the individual level with school-level disadvantage (unstandardized B (standard error)). The analysis was based on 57 schools.

	Victimized (N = 3932) *b* (SE)	Bully Perpetrators (N = 3932) *b* (SE)	Bully–Victims (N = 3932) *b* (SE)	Not Involved (N = 3932) *b* (SE)
Continuous predictor				
Intercept	0.37 (0.02) ***	0.01 (0.00) ***	0.07 (0.01) ***	0.53 (0.02) ***
School-level disadvantage (continuous)	0.00 (0.0)	0.00 (0.00)	0.002 (0.00) ***	0.00 (0.10)
Random Effects Intercept (SD)	0.05	0.01	0.03	0.07
ICC	0.01	0.003	0.013	0.021
Binary predictor				
Intercept	0.38 (0.01) ***	0.01 (0.00) ***	0.09 (0.01) ***	0.51 (0.02) ***
School-level disadvantage (dichotomized)	−0.04 (0.2) *	0.00 (0.00)	0.03 (0.01) *	0.00 (0.02)
Random Effects Intercept (SD)	0.04	0.01	0.05	0.08

Note: *** *p* < 0.001. * *p* < 0.05.

**Table 2:** Associations between mean scores for bully perpetrator, reinforcer, defender, and outsider roles (PRQ) and school-level disadvantage (unstandardized B (standard error)). The analysis was based on 57 schools.

	Bully Perpetrator (N = 4108) *b* (SE)	Reinforcer (N = 4258) *b* (SE)	Defender (N = 4179) *b* (SE)	Outsider (N = 3677) *b* (SE)
Continuous predictor				
Intercept	0.26 (0.03) ***	1.33 (0.03) ***	4.70 (0.06) ***	2.66 (0.04) ***
School-level disadvantage (continuous)	0.003 (0.00) *	0.00 (0.00)	−0.01 (0.00) *	0.00 (0.00)
Random Effects Intercept (SD)	0.10	0.13	0.25	0.16
ICC	0.017	0.022	0.026	0.019
Binary predictor				
Intercept	0.28 (0.03) ***	1.35 (0.03) ***	4.70 (0.06) ***	2.67 (0.04) ***
School-level disadvantage (dichotomized)	0.04 (0.04)	−0.03 (0.04)	−0.25 (0.08) **	−0.02 (0.06)
Random Effects Intercept (SD)	0.11	0.12	0.25	0.16

Note: *** *p* < 0.001. ** *p* < 0.01. * *p* < 0.05.

**Table 3:** Multilevel regression models examining the associations between bullying involvement (OBVQ) and emotional symptoms (TSDQ) (unstandardized B (SE)). Model 1 is unadjusted, while model 2 is adjusted for age and sex. Models 3 (unadjusted) and 4 (adjusted for age and sex, where ‘boy’ = 0 and ‘girl’ = 1) additionally examine for the association with school-level disadvantage. School N = 50 (7 schools were not included due to missing TSDQ data). The comparison category for bullying involvement was children ‘not involved’.

	Model 1 (N = 3302) *b* (SE)	Model 2 (N = 3275) *b* (SE)	Model 3 (N = 3302) *b* (SE)	Model 4 (N = 3275) *b* (SE)
Intercept	1.66 (0.11) ***	0.55 (0.38)	1.37 (0.16) ***	0.29 (0.40)
Bullying perpetration	0.24 (0.31)	0.38 (0.31)	1.16 (0.50) *	1.40 (0.50) **
Victimization	0.30 (0.09) ***	0.33 (0.09) ***	0.30 (0.12) *	0.31 (0.12) *
Bully–victim	0.46 (0.13) ***	0.56 (0.14) ***	0.25 (0.20)	0.35 (0.20)
Age		0.11 (0.04) **		0.11 (0.04) **
Sex		0.31 (0.08) ***		0.31 (0.08) ***
School-level disadvantage			0.02 (0.01) *	0.02 (0.01) *
School-level disadvantage x Bully perpetration			−0.05 (0.02) *	−0.06 (0.02) *
School-level disadvantage x Victimization			0.00 (0.01)	0.00 (0.00)
School-level disadvantage x Bully–victim			0.01 (0.01)	0.01 (0.01)
Random Effects Intercept (SD)	0.69	0.69	0.64	0.64
ICC	0.087	0.088	0.075	0.076

Note: *** *p* < 0.001. ** *p* < 0.01. * *p* < 0.05.

**Table 4:** Multilevel regression models examining the effects of bullying involvement (OBVQ) and externalizing problems (TSDQ) (unstandardized B (SE)). Model 1 is unadjusted, while model 2 is adjusted for age and sex. Models 3 (unadjusted) and 4 (adjusted for age and sex, where ‘boy’ = 0 and ‘girl’ = 1) additionally examine for the effect of school-level disadvantage. School N = 50. The comparison category for bullying involvement was children ‘not involved’.

	Model 1 (N = 3302) *b* (SE)	Model 2 (N = 3275) *b* (SE)	Model 3 (N = 3302) *b* (SE)	Model 4 (N = 3275) *b* (SE)
Intercept	1.41 (0.08) ***	1.92 (0.31) ***	1.26 (0.11) ***	1.74 (0.31) ***
Bullying perpetration	1.81 (0.27) ***	1.50 (0.26) ***	2.23 (0.43) ***	1.87 (0.42) ***
Victimization	0.42 (0.07) ***	0.41 (0.07) ***	0.35 (0.11) **	0.37 (0.10) ***
Bully–victim	1.61 (0.11) ***	1.44 (0.11) ***	1.40 (0.17) ***	1.25 (0.16) ***
Age		0.00 (0.03)		0.00 (0.04)
Sex		−1.02 (0.07) ***		−1.02 (0.07) ***
School-level disadvantage			0.01 (0.01)	0.01 (0.01) *
School-level disadvantage x Bully perpetration			−0.02 (0.02)	−0.02 (0.02)
School-level disadvantage x Victimization			0.00 (0.00)	0.00 (0.00)
School-level disadvantage x Bully–victim			0.01 (0.09)	0.01 (0.01)
Random Effects Intercept (SD)	0.45	0.44	0.41	0.39
ICC	0.053	0.054	0.043	0.042

Note: *** *p* < 0.001. ** *p* < 0.01. * *p* < 0.05.

**Table 5:** Multilevel regression models examining the effects of bullying role scores (PRQ) and emotional symptoms (TSDQ) (unstandardized B (SE)). Model 1 is unadjusted, while model 2 is adjusted for age and sex. Models 3 (unadjusted) and 4 (adjusted for age and sex, where ‘boy’ = 0 and ‘girl’ = 1) additionally examine for the effect of school-level disadvantage. School N = 50.

	Model 1 (N = 2665)	Model 2 (N = 2640)	Model 3 (N = 2665)	Model 4 (N = 2640)
Intercept	2.09 (0.21) ***	1.08 (0.44) *	1.72 (0.29) ***	0.71 (0.48)
Bully perpetrator	0.04 (0.06)	0.06 (0.06)	0.06 (0.10)	0.10 (0.10)
Reinforcer	0.08 (0.06)	0.08 (0.06)	0.17 (0.09) *	0.17 (0.09) *
Defender	−0.08 (0.03) **	−0.09 (0.03) **	−0.08 (0.04)	−0.09 (0.04) *
Outsider	−0.01 (0.03)	−0.02 (0.04)	−0.01 (0.04)	−0.03 (0.04)
Age		0.11 (0.05) *		0.11 (0.05) *
Sex		0.33 (0.09) ***		0.32 (0.09) ***
School-level disadvantage			0.02 (0.01)	0.02 (0.01)
School-level disadvantage x Bully perpetrator			0.00 (0.11)	−0.01 (0.01)
School-level disadvantage x Reinforcer			−0.02 (0.1)	−0.01 (0.01)
School-level disadvantage x Defender			0.00 (0.00)	0.00 (0.01)
School-level disadvantage x Outsider			0.00 (0.00)	0.00 (0.01)
Random Effects Intercept (SD)	0.71	0.72	0.67	0.68
ICC	0.09	0.091	0.081	0.083

Note: *** *p* < 0.001. ** *p* < 0.01. * *p* < 0.05.

**Table 6:** Multilevel regression models examining the effects of bullying role scores (PRQ) and externalizing problems (TSDQ) (unstandardized B (SE)). Model 1 is unadjusted, while model 2 is adjusted for age and sex. Models 3 (unadjusted) and 4 (adjusted for age and sex, where ‘boy’ = 0 and ‘girl’ = 1) additionally examine for the effect of school-level disadvantage. School N = 50.

	Model 1 (N = 2665)	Model 2 (N = 2640)	Model 3 (N = 2665)	Model 4 (N = 2640)
Intercept	2.24 (0.16) ***	2.54 (0.35) ***	2.11 (0.22) ***	2.39 (0.38) ***
Bully perpetrator	0.60 (0.05) ***	0.55 (0.05) ***	0.68(0.08) ***	0.58 (0.08) ***
Reinforcer	0.06 (0.05)	0.06 (0.05)	0.02 (0.07)	0.02 (0.07)
Defender	−0.10 (0.02) ***	−0.08 (0.02) **	−0.10 (0.04) **	−0.07 (0.04) *
Outsider	−0.11 (0.03) ***	−0.10 (0.03) **	−0.11 (0.03) ***	−0.10 (0.03) **
Age		0.00 (0.04)		−0.01 (0.04)
Sex		−0.93 (0.07) ***		−0.93 (0.07) ***
School-level disadvantage			0.01 (0.01)	0.01 (0.01)
School-level disadvantage x Bully Perpetrator			−0.01 (0.01)	0.00 (0.01)
School-level disadvantage x Reinforcer			0.01 (0.01)	0.01 (0.01)
School-level disadvantage x Defender			0.00 (0.01)	0.00 (0.00)
School-level disadvantage x Outsider			0.00 (0.01)	0.00 (0.00)
Random Effects Intercept (SD)	0.38	0.40	0.37	0.37
ICC	0.04	0.045	0.037	0.038

Note: *** *p* < 0.001. ** *p* < 0.01. * *p* < 0.05.

## Data Availability

The data presented in this study are available on request from the corresponding author. The data are not publicly available due to sensitive nature of the data.
